# The Desmoplakin Phenotype Spectrum: Is the Inflammation the “Fil Rouge” Linking Myocarditis, Arrhythmogenic Cardiomyopathy, and Uncommon Autoinflammatory Systemic Disease?

**DOI:** 10.3390/genes15091234

**Published:** 2024-09-22

**Authors:** Saverio D’Elia, Adriano Caputo, Francesco Natale, Enrica Pezzullo, Giuseppe Limongelli, Paolo Golino, Giovanni Cimmino, Francesco S. Loffredo

**Affiliations:** 1 Cardiology Unit, Azienda Ospedaliera Universitaria Luigi Vanvitelli, Piazza Miraglia 2, 80138 Napoli, Italy; saverio.delia@policliniconapoli.it; 2 Department of Translational Medical Sciences, Section of Cardiology, University of Campania Luigi Vanvitelli, 80131 Naples, Italy; dr.adrianocaputo@gmail.com (A.C.); giuseppe.limongelli@unicampania.it (G.L.); paolo.golino@unicampania.it (P.G.); francesco.loffredo@unicampania.it (F.S.L.); 3 Vanvitelli Cardiology and Intensive Care Unit, Monaldi Hospital, 80131 Naples, Italy; natalefrancesco@hotmail.com (F.N.); enrica.pezzullo@ospedalideicolli.it (E.P.); 4 Inherited and Rare Cardiovascular Diseases, Monaldi Hospital, 80131 Naples, Italy

**Keywords:** myocarditis, genetic, desmoplakin

## Abstract

Myocarditis is an inflammatory condition of cardiac tissue presenting significant variability in clinical manifestations and outcomes. Its etiology is diverse, encompassing infectious agents (primarily viruses, but also bacteria, protozoa, and helminths) and non-infectious factors (autoimmune responses, toxins, and drugs), though often the specific cause remains unidentified. Recent research has highlighted the potential role of genetic susceptibility in the development of myocarditis (and in some cases the development of inflammatory dilated cardiomyopathy, i.e., the condition in which there is chronic inflammation (>3 months) and left ventricular dysfunction\dilatation), with several studies indicating a correlation between myocarditis and genetic backgrounds. Notably, pathogenic genetic variants linked to dilated or arrhythmic cardiomyopathy are found in 8–16% of myocarditis patients. Genetic predispositions can lead to recurrent myocarditis and a higher incidence of ventricular arrhythmias and heart failure. Moreover, the presence of DSP mutations has been associated with distinct pathological patterns and clinical outcomes in arrhythmogenic cardiomyopathy (hot phases). The interplay between genetic factors and environmental triggers, such as viral infections and physical stress, is crucial in understanding the pathogenesis of myocarditis. Identifying these genetic markers can improve the diagnosis, risk stratification, and management of patients with myocarditis, potentially guiding tailored therapeutic interventions. This review aims to synthesize current knowledge on the genetic underpinnings of myocarditis, with an emphasis on desmoplakin-related arrhythmogenic cardiomyopathy, to enhance clinical understanding and inform future research directions.

## 1. Introduction

Myocarditis is, by definition, an inflammatory process affecting cardiac tissue. It represents an extremely heterogeneous condition, characterized by great variability in both clinical presentation and evolution [1]. From an etiological point of view, the causes can be infectious (mainly viruses, but also bacteria, protozoa, and helminths) as well as non-infectious (autoimmune, toxic, drugs), but often the cause of inflammation remains unknown [2]. Recently, the role of genetic susceptibility has been repeatedly raised in clinical and experimental studies in an attempt to understand why some individuals develop myocarditis while others, perhaps exposed to the same risk, do not [2]. In addition, it is still unclear how some individuals who develop myocardial inflammation recover without sequelae, while others may go through a chronic process of inflammation and ventricular dilatation by developing inflammatory dilated cardiomyopathy [2]. A body of literature is emerging suggesting a link between specific genotypes and myocarditis events and inflammatory cardiomyopathy [3]. Pathogenic variants of disease-causing genes for dilated or arrhythmic cardiomyopathy have been linked to the occurrence of myocarditis (up to 8–16%) [3]. Nevertheless, as proposed by Arbustini et al. [4], this topic needs to be carefully discussed to avoid the risk of turning an infectious into a genetic disease. Indeed, the distinction between genetic susceptibility and exclusive environmental factors that might aggregate within families can be difficult. In addition, especially in recent years, genetic evaluation is driving a change in the determination of residual risk after myocardial inflammatory processes. Desmoplakin (DSP) is an intracellular protein with the role of anchoring intermediate filaments to desmosomes in cardiomyocytes [5]. DSP mutation may result in recurrent left ventricular (LV) inflammation and myocardial injury, leading to myocardial fibrosis, dilated cardiomyopathy, or arrhythmogenic cardiomyopathy (ACM) [6,7]. Based on the available data, the role of DSP in myocarditis, DCM, and ACM is intriguing.

Myocarditis is still currently classified by the World Health Organization as a non-genetic disease [8]. The aim of this review is to describe the state of knowledge on the role of genetic background in myocarditis, with a specific focus on the clinical and molecular link/overlap with ACM, and in particular the major role played by DSP variants to explain this link. As described afterwards, other mutations in genes coding for proteins, especially cytoskeletal ones, may also underlie the development of myocarditis and subsequent heart disease, with mechanisms that are still unclear, but this will not be a specific subject of this review.

## 2. Literature Sources and Search Strategy

We performed a non-systematic review of the literature by applying the search strategy in different electronic databases (MEDLINE, EMBASE, Cochrane Register of Controlled Trials, and Web of Science). Original reports, meta-analyses, and review articles in peer-reviewed journals up to July 2024 regarding myocarditis, desmoplakin, autoimmunity, and genetic background were incorporated into the search strategy. The references of all identified articles were reviewed to look for additional papers of interest, to extrapolate the more recent available data on desmoplakin-related myocarditis, with a slight view of other possible genes involved.

## 3. Genetic Background of Myocarditis

Myocarditis has historically been considered to be an “unfortunate and random consequence of a viral infection” [9]. However, especially in recent years, we have assisted in a shift in this paradigm, due to the growing interest in determining whether certain genetic mutations could predispose individuals to cardiac inflammation, with or without association with pathological or physiological environmental stressor (viral infection, intense exercise, exposure to cardiotoxic molecules, or pregnancy). It is essential to highlight that myocarditis can progress to inflammatory cardiomyopathy when cardiac remodeling and heart dysfunction occur due to chronic inflammation [10]. The importance of investigating the genetic substrates of patients affected by myocarditis has been underlined by multiple authors, such as a recent retrospective multicenter study by Ammirati et al. [11], in which it was observed that individuals carrying a pathogenic mutation (89% DSP variant carriers) were more susceptible to recurrent myocarditis and ventricular arrhythmias than genotype-negative patients or individual not subjected to genetic testing (62.3% vs. 17.5% vs. 5.3% at 5 years, *p* < 0.0001). Additionally, 22% of the desmosomal gene variant group reported at least one relative with a history of myocarditis. Lota et al. identified dilatated cardiomyopathy (DCM)- or arrhythmogenic cardiomyopathy (ACM)-associated genetic variants in 8% of patient with acute myocarditis. In particular, in a cohort of 230 patients (male prevalence: 84%) diagnosed with acute myocarditis, a truncating variant of desmoplakin was present in 3.1% vs. 0.4% of controls (*p* = 0.001) and was characterized by normal left ventricular ejection fraction [12]. Similarly, in a recent meta-analysis by Monda et al. [13], considering an overall population of 586 individuals, a significant proportion of patients with myocarditis carried a pathogenic or likely pathogenic mutation associated with cardiomyopathies (24.7%). More specifically, sarcomeric mutations (mainly of the *TTN* or *MYH7* genes) were more frequently associated with complicated myocarditis (21.9%), while desmosomal protein mutations (foremost the *DSP* gene) were associated with an uncomplicated course (4.2%). Focusing on the *DSP* gene, its pathological variants have been isolated in both autosomal-dominant and autosomal-recessive forms of ACM, either with predominant left ventricular involvement (more frequent) or with right ventricular or biventricular involvement [14,15,16]. More specifically, missense mutations are generally located within the PKP/PKG and desmin-binding domains (N-terminal domain) [6] and seem to mostly affect protein stability, inter-domain contacts, and intra-protein interactions. Non-missense mutations (defined as frameshift, nonsense, or splice site variants that result in premature termination codons) are dispersed throughout the *DSP* gene and seem to result mostly in decreased localization of the protein in the intercalated disc (ID) [17,18]. More specifically, the *DSP* gene is particularly intolerant to truncating variants, which determine a loss of function of the protein and are more frequently associated with heart dysfunction [6,17,18,19].

Whereas the precise molecular mechanism that links DSP variants to ACM has not been fully elucidated, the evidence of inflammatory infiltrates in more than two-thirds of ACM heart samples suggests an important role of recurrent myocarditis as a driver in developing the cardiomyopathy phenotype [20]. A possible mechanism could be linked to dysregulation of the Wnt/β-catenin pathway. More specifically, *DSP* gene suppression in a cardiac cell line (HL-1 cells) and in knockout mice led to translocation of PKG from the desmosome to the nucleus, leading to inhibition of the genes belonging to the Wnt/β-catenin pathway, causing an increase in GSK3β and an upregulation of NF-κB activity, which could be determinants of inflammation, myocyte apoptosis, increased fibro-adipogenesis, and decreased myogenesis [21]. In addition, considering the case of recurrent myocarditis in two identical twin young athletes carrying a pathological DSP mutation [22], it has been hypothesized that certain DSP variants determine an increased susceptibility of the cardiac desmosome to mechanical stress, which could determine loss of intercellular connection, unmasking cardiomyocyte epitopes and subsequent formation of self-directed antibodies (anti-heart, anti-intercalated-disc, and anti-desmosome autoantibodies) responsible for the activation and maintenance of autoinflammatory processes.

## 4. Desmoplakin and Desmosome Structure

**Figure 1:**
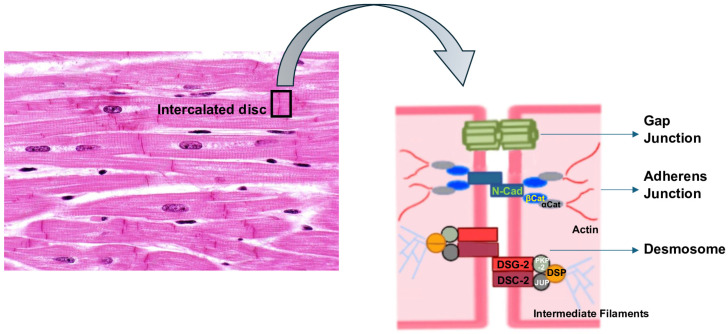
The intercalated disc (ID) structure. αCat: α-catenin; βCat: β-catenin; DSC-2: desmocollin-2; DSG-2: desmoglein-2; DSP: desmoplakin; JUP: plakoglobin; PKP-2: plakophillin-2; N-Cad: N-cadherin.

Cardiomyocytes are connected to each other through the intercalated disc (ID), a highly specialized structure that includes three types of intercellular contacts: adherens junctions, desmosomes and gap junctions [23], with desmosomes being involved in the crosstalk between adherens and gap junctions [24] (Figure 1).

In particular, a desmosome is a symmetrical protein complex of epithelia and cardiac muscle, specialized in ensuring strong adhesion between cells, and which participates in signaling pathways, influencing the transcriptional regulation of genes involved in proliferation, differentiation, and morphogenesis [25,26,27,28]. Its importance is testified by its highly preserved structure throughout the evolution process.

The products of three gene superfamilies assemble to form cardiomyocytes’ desmosomes:

- Plakins: desmoplakin (DSP).
- Desmosomal cadherins: desmoglein-2 (DSG-2) and desmocollin-2 (DSC-2).
- Armadillo/catenin family of nuclear and junctional proteins: plakoglobin (JUP) and placophillin-2 (PKP-2).

**Figure 2:**
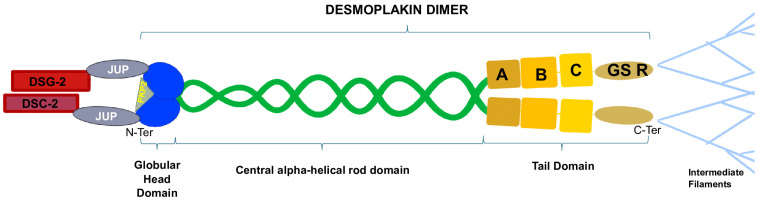
Desmoplakin is composed of an N-terminal globular head domain, a central α-helical rod domain, and a C-terminal tail domain. PKP-2 binds the globular head domain, stabilizing the DSP dimer, while JUP connects DSP to the transmembrane domain of the desmosome. The DSP C-terminal A-B-C plakin repeat domains and the glycine–serine–arginine-rich regions mediate the interactions between the desmosome and the intermediate filaments.

More specifically, DSG-2 and DSC-2 are transmembrane proteins, with their cytoplasmic domains residing in the desmosomal plaque, and their extracellular domains form dimers [29]. DSP, JUP, and PKP-2 are located in the intracellular compartment and stabilize the desmosomal structure by linking membrane components with intermediate filaments (IFs) [30], acting as a force transducer between the two structures. DSP has a tripartite structure that includes the amino-terminal (N-terminal) globular head domain (Head) that mediates protein–protein interactions, a central α-helical rod domain, and a carboxy-terminal (C-terminal) tail domain. The C-tail contains three plakin repeat domains (PRDs A, B, C) and a glycine–serine–arginine (GSR)-rich region that is thought to regulate DSP’s binding to IFs [17] (Figure 2). JUP binds the cadherin cytoplasmic tails and N-terminal plakin domain of DSP dimers, while PKP-2 connects the N-terminal domains of DSP with each other [31]. Ultrastructural alterations of DSP can cause multiple pathological phenotypes, possibly due to the disruption of the integrity of the desmosome, which weakens the mechanical coupling between cardiomyocytes. This may lead to a mechanical failure, activating innate immunity and, eventually, myocardial remodeling and fibrosis [8].

## 5. Arrhythmogenic Cardiomyopathy (ACM)

Arrhythmogenic cardiomyopathy (ACM) is a genetically determined myocardial disease that is characterized by necrosis of myocytes with fibrous replacement and the presence of ventricular arrhythmias, which can also lead to sudden cardiac death (SCD), especially in the young. In about 50% of cases, a causative genetic variant can be identified, which in most cases involves genes encoding for desmosomal proteins, although some non-desmosomal disease genes have been identified in recent years [32,33].

After early descriptions that considered the disease to be limited to the right ventricle (RV), contrast-enhanced cardiac magnetic resonance imaging (CE-CMR) studies have shown a frequent involvement of the left ventricle (LV) [34]. New diagnostic criteria, the “Padua criteria”, were proposed in 2020 to replace the previous 2010 International Task Force (TF) criteria [35]. The main changes were the increased weight given to cardiac MRI for the identification of tissue abnormalities and the introduction of criteria for defining left ventricular involvement, either isolated (left-dominant variant) or associated with that of the right ventricle (biventricular variant). One relevant finding is that, since the earliest descriptions of the disease, inflammatory cell infiltrates have been reported in endomyocardial biopsy, heart autopsy, and heart transplant specimens from ACM patients [32,33]. In a study by Corrado et al., including 42 patients (some of whom had received heart transplants), inflammatory infiltrates were reported in up to 75% of human hearts analyzed [36]. In addition, episodes of chest pain accompanied by electrocardiographic changes and troponin release consistent with acute myocarditis, termed “hot phases”, have been described [37]. In some cases, these episodes may be the initial presentation of ACM and enter the differential diagnosis with acute myocarditis [38]. The diagnosis of ALVC, in the absence of RV involvement, requires, in addition to the identification of an LV phenotype compatible with ACM, the presence of a pathogenic mutation. The specificity of structural and morphofunctional abnormalities of the LV is reduced because of the overlap of manifestations with other diseases, such as DCM, myocarditis, or sarcoidosis. Arrhythmogenic cardiomyopathy is indeed a genetically heterogeneous disorder. A list of genes in which mutations are known to cause this condition is shown in Table 1.

The discovery of the genetic basis of Naxos disease, characterized by palmoplantar keratoderma, woolly hair, and ACM, led to the identification of desmosome mutations as a cause of ACM. Mutations in the *JUP* gene were initially linked to Naxos disease, while similar discoveries in South America identified mutations in the *DSP* gene as a cause of Carvajal syndrome. Subsequently, a dominant DSP mutation was discovered to cause non-syndromic AC. Further research has found that mutations in other desmosomal genes, such as *PKP2*, *DSG2*, and *DSC2*, are also common causes of ACM. Key proteins such as α-T-catenin and N-cadherin, involved in cell adhesion, have been linked to AC through mutations in their respective genes (*CTNNA3* and *CDH2*). Nuclear membranes are also involved in AC, specifically through mutations in the *LMNA* gene, which encodes lamin A/C, and the *TMEM43* gene. These mutations are associated with various forms of cardiomyopathy, often involving both ventricles, and can also lead to other conditions such as muscular dystrophy [39].

In particular the “hot phase” represents an uncommon clinical presentation of ACM, often occurring in pediatric patients and carriers of desmoplakin gene mutations [40]. Tissue characterization, family history, and genetic testing are key diagnostic tools for differential diagnosis [38]. It is interesting to mention that, in patients with DSP mutations and recurrent myocarditis, intense physical activity has been described as another potential trigger [22]. Smith et al. [6] proposed the term “desmoplakin cardiomyopathy”, which describes a clinical phenotype characterized by a large amount of left ventricular fibrosis (typically assessed with cardiac magnetic resonance imaging and characterized by ring-like LGE patterns) [41] with normal left cardiac function, episodes of myocardial necrosis, and a significant degree of electrical instability.

Bauce et al., who reported a series of 38 individuals carrying DSP genetic variants from four different ACM families, concluded that familial ACM due to DSP variants was characterized by a high incidence of sudden cardiac death (SCD), and that left ventricular (LV) involvement was not a rare feature. In addition, chest pain associated with ST segment elevation on baseline electrocardiogram (ECG), along with myocardial enzyme release, was observed in two cases, in the context of angiographically normal coronary arteries [42].

According to the authors, desmoplakin cardiomyopathy should be considered in the differential diagnosis of acute myocardial inflammatory syndromes, including myocarditis and sarcoidosis. This thesis is supported by the histological study of explanted hearts with arrhythmogenic cardiomyopathy, where hearts with DSP cardiomyopathy showed a distinct pattern of pathological fibrosis when compared to hearts with typical arrhythmogenic right ventricular cardiomyopathy [43].

Ammirati et al. [11] described a subpopulation of patients with acute myocarditis and evidence of mutation in desmoplakin genes. Patients with acute myocarditis and desmoplakin mutations were selected for the absence of major or minor imaging criteria for arrhythmogenic cardiomyopathy according to the Task Force criteria revised in 2010. These patients had an initial presentation most commonly characterized by chest pain as a major symptom and had preserved or mildly reduced biventricular systolic function. In general, these features should suggest a benign long-term prognosis, although this population presented a higher incidence of adverse events at follow-up.

This evidence underlines the importance of differential diagnosis between acute myocarditis, ACM, and desmoplakin cardiomyopathy, although this can be difficult due to the genetic and phenotypic overlap of these conditions.

## 6. Discussion: Unveiling the Desmoplakin Disease Spectrum

Acute myocarditis is an important cause of heart failure and of the development of cardiomyopathy in young adults and children. Although the risk factors are not completely understood, myocarditis, especially when recurrent, has been linked to some pathogenic variants in cardiac genes that are traditionally associated with cardiomyopathies. The role of genetics in both viral susceptibility and prognosis should improve the mechanistic understanding of the pathways leading to myocarditis, identifying patients who may develop chronic inflammatory cardiomyopathy (the condition in which there is chronic inflammation lasting more than 3 months and left ventricular dysfunction\dilatation). In addition, the presence of certain DSP variants may be useful in recognizing higher-risk phenotypes in patients who will develop arrhythmias and heart failure at follow-up [11]. Moreover, DSP mutation also seems to identify patients at high risk of recurrent myocarditis [44]. Data from the published literature do not entirely support the notion that myocarditis associated with genetic variants is a condition determined by increased susceptibility to pathogens or certain phenotypic expressions, often initial signs of cardiomyopathy. In a study by Bassetto et al. [37], seventeen patients hospitalized for chest pain or acute heart failure underwent both endomyocardial biopsy (EMB) and genetic testing: histological detection of apoptosis at EMB was frequent (77% of cases), and all patients who tested positive for pathogenic/likely pathogenic variants showed apoptosis at EMB. In patients with apoptosis at EMB, the left ventricular ejection fraction was lower at the first clinical presentation and improved during follow-up with anti-neurohormonal therapy. These patients were framed as cases of cardiomyopathy during an acute episode of troponin release. In nine patients (52% of the total cohort), apoptosis was isolated, whereas the remaining four patients (23% of the total cohort) showed significant inflammation concomitant to apoptosis. Inflammation with no signs of apoptosis was found in only two cases (12% of the total cohort). There was no evidence of necrosis or fibrofatty tissue at EMB. In a case report by Catapano et al. [45], a patient with fulminant myocarditis underwent DNA sequencing for 174 common cardiomyopathy-causing genes, with next-generation sequencing detecting pathogenic heterozygous variants in the form of nonsense mutations of the desmoplakin (DSP) gene associated with an unusual and severe presentation of adult-onset Still’s disease. We speculate that the presence of a pathogenic variant may facilitate myocardial injury in systemic inflammatory diseases, although specific studies are required.

Specifically, for patients with DSP mutations, the inflammatory burden seems to be a determinant of progression, as indicated by some case reports [6,18,46]. It is suggested that DSP cardiomyopathy is characterized by intermittent myocardial inflammatory episodes that are clinically similar to myocarditis or sarcoidosis, as shown by a case series of DSP cardiomyopathy where MRI and positron emission tomography evaluations clearly revealed the underlying inflammatory pattern [6]. These episodes occur even in the presence of normal systolic function, suggesting that myocardial injury and fibrosis might be an early step in DSP cardiomyopathy. However, the frequency of this finding and its association with disease stage remain unclear. Based on the published literature, acute myocardial inflammatory episodes might be the proximal cause of myocardial fibrosis and progressive dysfunction in the presence of DSP mutations [6]. Modulation of inflammatory pathways might be a novel therapeutic strategy for desmosomal-mediated cardiomyopathy, as recently shown in an animal model carrying homozygous mutations in DSG2 [47].

**Figure 3:**
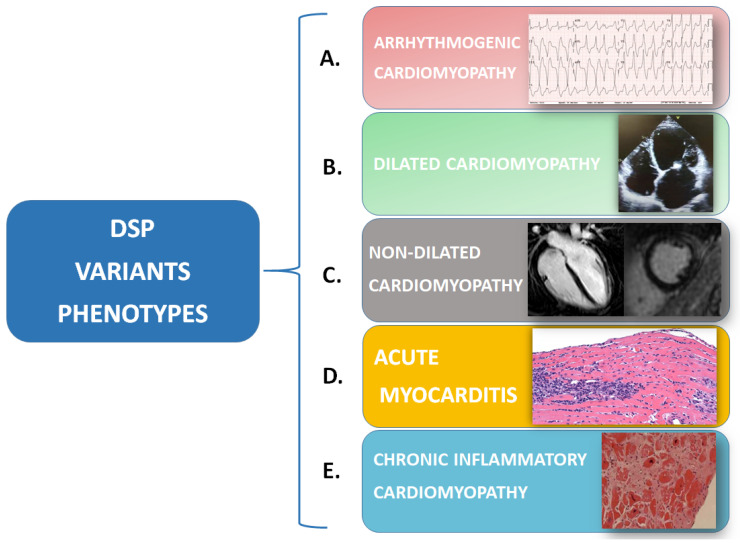
Desmoplakin variants can cause a spectrum of phenotypes, which can sometimes coexist at the same moment or can be expressed in the same patient at different times: (**A**) Arrhythmogenic cardiomyopathy is characterized by EKG anomalies and a high arrhythmic burden (i.e., right bundle-branch block morphology ventricular tachycardia) that can be associated with morphological/functional anomalies of one or both ventricles. (**B**) Dilated cardiomyopathy is characterized by isolated or biventricular dilatation and global or regional systolic dysfunction. (**C**) Non-dilated cardiomyopathy is defined by the presence of morphological anomalies (i.e., ring-like LGE at CMR) or isolated systolic dysfunction of one or both ventricles. (**D**) In acute myocarditis, endomyocardial biopsy shows a white blood cell infiltration of the myocardium that can be associated with anomalies of cardiomyocytes, apoptosis, and/or signs of necrosis. (**E**) In chronic inflammatory cardiomyopathy endomyocardial biopsy samples, fibrosis predominates over white blood cell infiltration.

Thus, the “fil rouge” of inflammation may be the hidden link between different diseases, such as myocarditis—as a possible early-stage arrhythmogenic cardiomyopathy—and rare systemic diseases under the umbrella of the “desmoplakin disease spectrum”, including dilated or non-dilated cardiomyopathy, chronic inflammatory cardiomyopathy, and ACM (Figure 3). To date, it remains to be clarified whether an inflammatory trigger is the “primum movens” that determines cardiomyocytes’ damage, necrosis, and the consequent repair process with fibrofatty infiltration, or if inflammation is rather a reactive phenomenon following myocyte loss.

It is pathophysiologically straightforward to think that an alteration in the proteins that are part of the desmosome as the desmoplakin complex, due to a variety of stimuli (i.e., parietal stress from exercise, hypertension, toxicity), can trigger the release of pro-inflammatory molecules that initiate and perpetuate an inflammatory response, resulting in myocardial damage. The same stimuli would indeed not produce the same effects in individuals not carrying the mutation. Based on the current literature, there is ambiguous interpretation of the data. However, a myocarditis episode, whether infectious or not, and especially if relapsing or occurring within a family in which there have been other members with myocarditis or cardiomyopathies, warrants genetic study both in a prognostic manner and to unveil the presence of possible genetically imprinted cardiomyopathies [13]. The possibility of easily performing DNA sequencing may represent a novel diagnostic marker to identify patients who present with symptoms of myocarditis and may benefit from specific follow-ups. Furthermore, while the presence of pathogenic genetic variants cannot, at the moment, be corrected, discovering the relevance of genetic background in myocarditis may help to perform clinical studies to evaluate the prognostic impact of drugs that may modulate the inflammatory response.

A practical key message is to raise awareness that mutations of genes associated with cardiomyopathies may initially present as myocarditis, often relapsing and associated with a mild clinical presentation. This may drive the clinician to perform additional diagnostic evaluation [48].

It is intuitive to think that the recurrence of symptoms, sometimes associated with massive troponin release, may be ascribed to exposure to causative factors (e.g., viral infection), or to a mechanical breakdown of the cell membrane with subsequent immune activation.

At the moment, genetic analysis is not recommended routinely in patients with myocarditis.

The awareness that inflammation is a veiled or overt manifestation of many cardiomyopathies, and that the first manifestation of a cardiomyopathy may be myocarditis, suggests a change in the current diagnostic and clinical thinking process.

As in an investigation process, putting together the family history (positive history of cardiomyopathies, sudden cardiac death), the personal history (recurrences of myocarditis), simple diagnostic tests such as ECGs, and more complex imaging tests such as magnetic resonance imaging, in which characteristic LGE patterns (ring-like pattern) may be present, the addition of genetic analysis could be a fundamental element to allow a correct classification and an appropriate follow-up of these patients. To date, however, genetic testing does not allow any specific treatment. Indeed, there are weak data suggesting that modulating inflammation using immunosuppressive drugs may have an impact on the disease course, and this will require extensive studies.

On the other hand, early implantation of a defibrillator in cases of recurrent myocarditis with even non-complex arrhythmias in patients with desmoplakin mutations could be a fundamental move quoad vitam in most selected cases, and especially when there is a family history of sudden cardiac death or life-threatening arrhythmias [11].

## 7. Conclusions

Cardiac disease phenotypes presenting as myocarditis or as a cardiomyopathy associated with an inflammatory burden bearing mutations of desmosomal proteins, including desmoplakin, are currently heterogeneously classified as inflammatory cardiomyopathies, as ACM, or as relapsing myocarditis with unfavorable evolution (fatal arrhythmias or heart failure). While still complex, because the prevalence to date is not entirely clear and these patients are often treated as simple myocarditis cases without further investigation in tertiary centers, a novel uniform classification and taxonomy may facilitate research and patients’ treatment.

The emerging trend is that desmoplakin mutations may underlie diseases that are currently classified heterogeneously but are actually the same condition, presenting with a clinical spectrum that may range from myocarditis as an initiating manifestation in some subjects, to ventricular arrhythmias in others without clinical signs of myocarditis, but with an underlying inflammatory component, possibly due to cellular disruption\apoptosis and subsequent inflammatory activation.

In the immediate future, standardizing taxonomy, pooling similar mutations, and clinical evaluation of this heterogeneous group of patients will be crucial to optimize follow-up and therapies. Ongoing research, including the use of gene editing, may represent a thrilling opportunity to cure these diseases.

## Figures and Tables

**Table 1:** List of genes associated with ACM. Modified from the work of Corrado et al. [39].

Gene	Encoded Protein	Subcellular Localization	Chromosomal Locus
*JUP*	Junction plakoglobin	Desmosome	17q21.2
*DSP*	Desmoplakin	Desmosome	6p24.3
*PKP2*	Plakophilin-2	Desmosome	12p11.21
*DSG2*	Desmoglein-2	Desmosome	18q12.1
*DSC2*	Desmocollin-2	Desmosome	18q12.1
*TMEM43*	Transmembrane protein 43 (luma)	Nuclear envelope	3p25.1
*LMNA*	Lamin A/C	Nuclear envelope	1q22
*DES*	Desmin	Intermediate filament	2q35
*CTNNA3*	α-T-catenin	Area composita	10q21.3
*PLN*	Phospholamban	SERCA	6q22.31
*TGFB3*	Transforming growth factor-3	Growth factor	14q24.3
*TTN*	Titin	Sarcomere	2q31.2
*SCN5A*	Sodium voltage-gated channel α subunit 5 (NaV1.5)	Sodium channel	3p22.2
*CDH2*	Cadherin C	Area composita	18q12.1
